# Common and Distinct Genetic Properties of ESCRT-II Components in *Drosophila*

**DOI:** 10.1371/journal.pone.0004165

**Published:** 2009-01-09

**Authors:** Hans-Martin Herz, Sarah E. Woodfield, Zhihong Chen, Clare Bolduc, Andreas Bergmann

**Affiliations:** 1 Department of Biochemistry and Molecular Biology, The Genes & Development Graduate Program, The University of Texas M. D. Anderson Cancer Center, Houston, Texas, United States of America; 2 Baylor College of Medicine, Graduate Program in Developmental Biology, Houston, Texas, United States of America; Institut Pasteur, France

## Abstract

**Background:**

Genetic studies in yeast have identified class E *vps* genes that form the ESCRT complexes required for protein sorting at the early endosome. In *Drosophila*, mutations of the ESCRT-II component *vps25* cause endosomal defects leading to accumulation of Notch protein and increased Notch pathway activity. These endosomal and signaling defects are thought to account for several phenotypes. Depending on the developmental context, two different types of overgrowth can be detected. Tissue predominantly mutant for *vps25* displays neoplastic tumor characteristics. In contrast, *vps25* mutant clones in a wild-type background trigger hyperplastic overgrowth in a non-autonomous manner. In addition, *vps25* mutant clones also promote apoptotic resistance in a non-autonomous manner.

**Principal Findings:**

Here, we genetically characterize the remaining ESCRT-II components *vps22* and *vps36*. Like *vps25*, mutants of *vps22* and *vps36* display endosomal defects, accumulate Notch protein and – when the tissue is predominantly mutant – show neoplastic tumor characteristics. However, despite these common phenotypes, they have distinct non-autonomous phenotypes. While *vps22* mutations cause strong non-autonomous overgrowth, they do not affect apoptotic resistance. In contrast, *vps36* mutations increase apoptotic resistance, but have little effect on non-autonomous proliferation. Further characterization reveals that although all ESCRT-II mutants accumulate Notch protein, only *vps22* and *vps25* mutations trigger Notch activity.

**Conclusions/Significance:**

The ESCRT-II components *vps22*, *vps25* and *vps36* display common and distinct genetic properties. Our data redefine the role of Notch for hyperplastic and neoplastic overgrowth in these mutants. While Notch is required for hyperplastic growth, it appears to be dispensable for neoplastic transformation.

## Introduction

Appropriate cell/cell signaling requires both coordinated activation and inactivation of cell surface signaling receptors. Usually, the receptors are activated by ligand binding upon which they induce an intracellular response including ubiquitination of the receptor which provides the signal for receptor internalization by endocytosis [1]–[3]. Endocytosis also controls the steady-state levels of cell surface receptors independently of ligand occupation. After endocytosis, the cell surface receptors are present at the early endosome. Because the intracellular domain of activated signaling receptors is exposed to the cytosol, the receptors are still able to signal. In fact, signaling from the endosomal location appears to be the preferred mode of several signaling pathways as it brings the receptor in close proximity to intracellular signaling complexes [4]–[8]. To fully inactivate the signaling receptors, a second form of internalization at the limiting membrane of the early endosome is necessary to form the multi-vesicular body (MVB) [3], [9]–[14]. In the MVB, the receptors are completely detached from the cytosol and stop signaling. Finally, the MVB fuses with lysosomes for proteolytic degradation.

Genetic studies in yeast have identified fifteen class E *vps* (vacuolar protein sorting) genes required for MVB formation [15]. These genes encode the components of four ESCRT (Endosomal Sorting Complex Required for Transport) protein complexes (reviewed by [3], [9]). Hrs (Vps27) and STAM (Hse1) form ESCRT-0, which initiates the recruitment of the signaling receptor (the cargo) to the early endosome and delivers it to ESCRT-I. From there, the cargo is transferred to ESCRT-II and then to ESCRT-III. At ESCRT-III, the receptors are internalized into MVBs [3], [9]. Loss of class E *vps* function in yeast leads to accumulation of ubiquitinated proteins on the limiting membrane of enlarged endosomes [12]. Biochemical studies in mammalian cells have revealed a similar function for endosomal protein sorting [3], [9].

The phenotypic consequences of loss of class E *vps* genes in the context of a multi-cellular organism have just recently been unveiled. In *Drosophila*, mutants in *hrs*, *erupted* (*ept*, encoding the ESCRT-I component *vps23*) and *vps25* (a component of ESCRT-II) have recently been described. These mutants are characterized by enlarged endosomes which contain increased protein levels of Notch, Delta, EGFR, Patched, Smoothened, and Thickveins (the *Drosophila* TGFβ type 1 receptor) [16]–[21]. Despite these common endosomal defects, *hrs*, *ept* and *vps25* display different phenotypes at the organismal level. While *hrs* mosaics do not display any obvious adult phenotypes, *ept* and *vps25* mosaics are characterized by overgrown adult eyes and heads, and overgrown larval imaginal discs due to hyperplastic proliferation. Hyperplastic proliferation refers to increased proliferation and overgrowth; however, hyperplastic cells still maintain epithelial polarity and will eventually stop proliferating [22]. Interestingly, this hyperplastic growth does not occur in *ept* and *vps25* mutant tissue itself. Instead, it occurs in wild-type cells immediately abutting the mutant tissue [18]–[21]. This non-autonomous hyperplastic proliferation is caused by increased Notch activity at the *ept* and *vps25* endosomes which stimulates neighboring cells to undergo proliferation by activating the Jak/STAT pathway [23]–[25]. Increased Notch activity has not been observed in *hrs* mutants despite the accumulation of Notch protein, explaining the lack of hyperplastic overgrowth in *hrs* mutants.

In addition to non-autonomous hyperplastic growth in genetic mosaics, *ept* and *vps25* mutations can cause neoplastic overgrowth. Neoplastic cells lose epithelial polarity and fail to stop proliferating giving rise to significant overgrowth [22]. *ept* and *vps25* mutants show neoplastic overgrowth if almost the entire imaginal disc is mutant [18], [20], [26]. Neoplastic overgrowth can also be induced in *vps25* mosaic tissue, if apoptosis is blocked in *vps25* mutant cells [19], [21]. Under both conditions, neoplastic growth occurs in an autonomous manner, i.e. in the mutant tissue [19], [21]. These findings were significant for a better understanding of tumor formation caused by inactivation of *Tsg101* (tumor susceptibility gene 101), the human *vps23* homolog, which has been implicated in cervical, breast, prostate and gastrointestinal cancers [27]–[31].

In addition, although *vps25* mutant cells undergo apoptosis, before they die they can increase the apoptotic resistance of neighboring cells through up-regulation of the apoptosis inhibitor Diap1 (*Drosophila* Inhibitor of Apoptosis Protein 1) [21].

Except for *vps25*, a genetic analysis of the ESCRT-II components for endosomal protein sorting in metazoan organisms has not been reported. Here, we characterize and compare the mutant phenotypes of the individual components of the ESCRT-II complex, *vps22* (also called *larsen* [32]), *vps25* and *vps36* in *Drosophila*. The ESCRT-II complex is a heterotetramer composed of two Vps25 subunits, and one subunit each of Vps22 and Vps36 [33], [34]. We show that mutant cells of the three ESCRT-II components display endosomal defects and accumulate Notch protein. Moreover, imaginal discs predominantly mutant for the three ESCRT-II components show characteristics of neoplastic tissue growth. However, despite these common defects, the phenotypic consequences of loss of *vps22*, *vps25* and *vps36* in mosaic animals are distinct. *vps22* and *vps25*, but not *vps36* mosaics show non-autonomous hyperplastic growth. In contrast, *vps25* and *vps36*, but not *vps22* mosaics strongly increase apoptotic resistance. We demonstrate that these differences are caused by selective Notch activation. *vps22* and *vps25* clones display high Notch signaling activity, while *vps36* clones do not, suggesting that hyperplastic growth depends on Notch signaling. However, neoplastic growth may be independent of Notch signaling. Thus, despite their intimate physical relationship, the individual ESCRT-II components are genetically not equivalent.

## Results

### Common phenotypes I: ESCRT-II mutants contain enlarged endosomes accumulating ubiquitinated proteins

**Figure 1:**
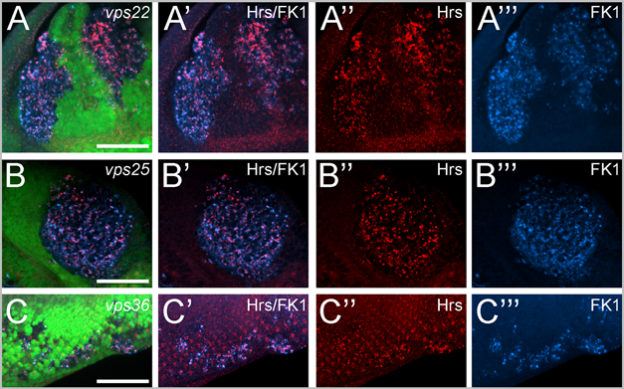
Mutant clones of ESCRT-II components display endosomal defects and accumulate ubiquitinated proteins. Shown are eye imaginal discs of 3rd instar larvae mosaic for ESCRT-II mutants. Mutant clones are marked by the absence of GFP. Mutant clones of ESCRT-II components show abnormal accumulation of the early endosomal marker Hrs and accumulation of ubiquitin-conjugated proteins as visualized by the FK1 antibody. Hrs and ubiquitin-conjugated proteins accumulate in foci which frequently co-localize. Scale bars represent 50 µm. (A,B,C) GFP/Hrs/FK1 (green/red/blue) co-labelings of (A) *vps22^5F8-3^*, (B) *vps25^N55^* and (C) *vps36^L5212^* eye mosaics. (A′,B′,C′) Hrs/FK1 (red/blue) co-labelings of (A′) *vps22^5F8-3^*, (B′) *vps25^N55^* and (C′) *vps36^L5212^* eye mosaics. (A″,B″,C″) Hrs labeling of (A″) *vps22^5F8-3^*, (B″) *vps25^N55^* and (C″) *vps36^L5212^* eye mosaics. (A′″,B′″,C′″) FK1 labeling of (A′″) *vps22^5F8-3^*, (B′″) *vps25^N55^* and (C′″) *vps36^L5212^* eye mosaics. Genotypes: (A) *eyFlp* ; *FRT82B vps22^5F8-3^*/*FRT82B* P[*ubi-GFP*]. (B) *eyFlp*; *FRT42D vps25^N55^*/*FRT42D* P[*ubi-GFP*]. (C) *eyFlp* ; *vps36^L5212^ FRT2A*/P[*ubi-GFP*] *FRT2A*.

Because *vps25* mutants in *Drosophila* are characterized by enlarged early endosomes [19]–[21] (see also Fig. 1B″), we tested whether mutants in the other two ESCRT-II components also contain abnormal endosomes. As endosomal marker we used an antibody raised against Hrs [17]. Mutant clones of *vps22* and *vps36* in eye imaginal discs contain enlarged Hrs-positive particles, representing abnormal early endosomes (Fig. 1A″,C″).

**Figure 2:**
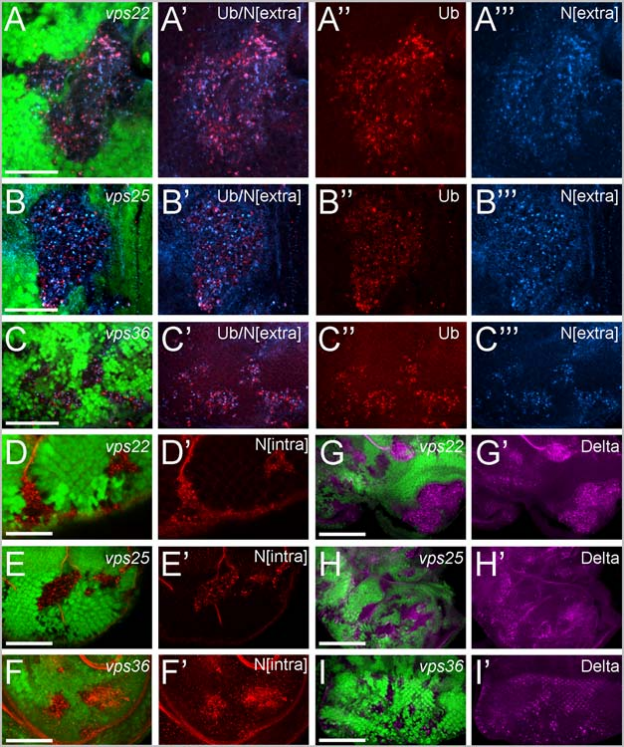
Accumulation of Notch and Delta proteins in clones of ESCRT-II mutants. (A,B,C) Areas of increased Ubiquitin (Ub) immunoreactivity frequently co-localize with areas containing accumulated levels of the Notch receptor (antibody against the extracellular domain of Notch, N[extra]) in ESCRT-II mutant tissue. GFP/Ubiquitin/N^extra^ (green/red/blue) co-labelings of (A) *vps22^5F8-3^*, (B) *vps25^N55^* and (C) *vps36^L5212^* eye mosaics. Scale bars represent 50 µm. (A′,B′,C′) Ubiquitin/N^extra^ (red/blue) co-labeling of (A′) *vps22^5F8-3^*, (B′) *vps25^N55^* and (C′) *vps36^L5212^* eye mosaics. (A″,B″,C″) Ubiquitin labeling of (A″) *vps22^5F8-3^*, (B″) *vps25^N55^* and (C″) *vps36^L5212^* eye mosaics. (A′″,B′″,C′″) N^extra^ labeling of (A′″) *vps22^5F8-3^*, (B′″) *vps25^N55^* and (C′″) *vps36^L5212^* eye mosaics. (D,E,F) Accumulation of Notch protein using an antibody detecting the intracellular domain of Notch, N[intra]). GFP/N^intra^ (green, red) co-labelings of (D) *vps22^5F8-3^*, (E) *vps25^N55^* and (F) *vps36^L5212^* eye mosaics. Scale bars represent 100 µm (D′,E′,F′) N^intra^ (red) labeling of (D′) *vps22^5F8-3^*, (E′) *vps25^N55^* and (F′) *vps36^L5212^* eye mosaics. (G,H,I) The Notch ligand Delta accumulates in clones of ESCRT-II mutants. GFP/Delta (green/magenta) co-labeling of (G) *vps22^5F8-3^*, (H) *vps25^N55^* and (I) *vps36^L5212^* eye mosaics. Scale bars represent 100 µm (G′,H′,I′) Delta labeling of (G′) *vps22^5F8-3^*, (H′) *vps25^N55^* and (I′) *vps36^L5212^* eye mosaics. Genotypes: (A,D,G) *eyFlp*; *FRT82B vps22^5F8-3^/FRT82B P[ubi-GFP]*. (B,E,H) *eyFlp* ; *FRT42D vps25^N55^/FRT42D P[ubi-GFP]*. (C,F,I) *eyFlp* ; *vps36^L5212^ FRT2A/P[ubi-GFP] FRT2A*.

The FK1 and FK2 antibodies recognize ubiquitin-conjugated proteins, but not unconjugated ubiquitin [35]–[37]. Using the FK antibodies, we found that the enlarged Hrs-positive particles accumulate ubiquitin-conjugated proteins (shown for FK1 in Fig. 1A′″–C′″). Similar data were obtained for FK2 antibody (data not shown). In addition, an antibody that recognizes both conjugated and unconjugated ubiquitin also detects increased abundance of ubiquitin in *vps22*, *vps25* and *vps36* mutant clones (Fig. 2A″, C″). Thus, *vps22*, *vps25*, and *vps36* mutant cells contain abnormally large early endosomes that accumulate ubiquitin-conjugated proteins.

### Common phenotypes II: Accumulation of Notch and Delta proteins ESCRT-II mutant clones

It has been reported that *vps25* mutants accumulate Notch protein in endosomes [19]–[21] (see also Fig. 2B,E). Thus, we analyzed *vps22* and *vps36* mutants for accumulation of Notch protein. Consistently, *vps22* and *vps36* mutant clones accumulate Notch protein in punctate particles that, due to their colocalization with ubiquitylated proteins, correspond to enlarged endosomes (Fig. 2A,C,D,F). This was found using antibodies that recognize both the extracellular domain of Notch (Fig. 2A′″–C′″) and the intracellular domain of Notch (Fig. 2D′–F′). In addition, an antibody raised against the Notch-ligand Delta also detects increased abundance of Delta protein in mutant clones (Fig. 2G′–I′).

### Common phenotypes III: ESCRT-II mutant clones are apoptotic

**Figure 3:**
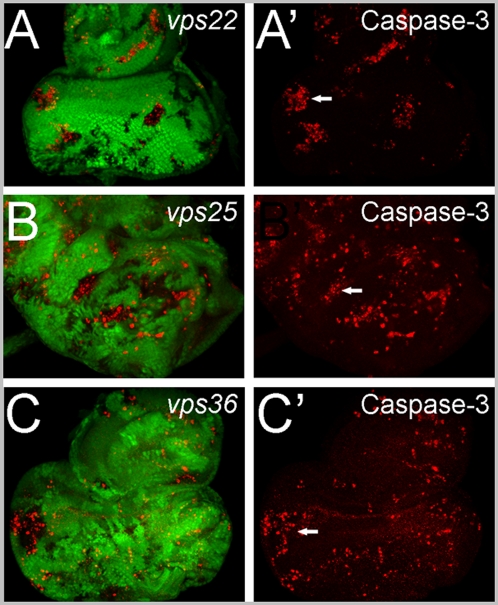
Apoptosis phenotype of ESCRT-II mutants. Labeling of *vps22*, *vps25* and *vps36* eye-antennal imaginal discs with cleaved Caspase-3 antibody as apoptotic marker. Arrows in (A′), (B′) and (C′) point to one representative clone in each panel containing increased caspase-3 activity. (A–C) GFP/Caspase-3 (green/red) co-labelings of (A) *vps22^5F8-3^*, (B) *vps25^N55^* and (C) *vps36^L5212^* eye mosaics. (A′–C′) Caspase-3 labeling (red) of (A′) *vps22^5F8-3^*, (B′) *vps25^N55^* and (C′) *vps36^L5212^* eye mosaics. Genotypes: (A) *eyFlp* ; *FRT82B vps22^5F8-3^/FRT82B P[ubi-GFP]*. (B) *eyFlp* ; *FRT42D vps25^N55^/FRT42D P[ubi-GFP]*. (C) *eyFlp* ; *vps36^L5212^ FRT2A/P[ubi-GFP] FRT2A*.

*vps25* mutant clones in eye imaginal discs are extremely apoptotic [19]–[21] (see also Fig. 3B). To test whether this applies to *vps22* and *vps36* mutant clones, we performed immunolabeling using an antibody that recognizes the cleaved and thus activated form of Caspase-3 [38]. In 3rd instar eye imaginal discs of wild-type larvae, apoptotic cell death does not occur [39], [40]. However, *vps22* and *vps36* clones contain increased caspase activity (Fig. 3A,C). Thus, as for *vps25*, loss of *vps22* and *vps36* causes the apoptotic death of the affected cells.

### Common phenotypes IV: imaginal discs predominantly mutant for ESCRT-II show disorganized cellular architecture and overgrowth

**Figure 4:**
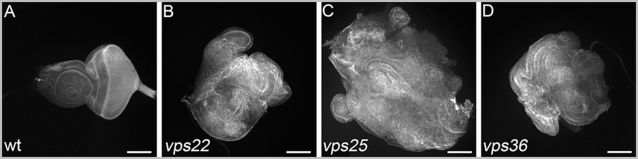
Eye discs predominantly mutant for ESCRT-II are overgrown and lose cellular architecture. All discs are labeled for phalloidin and were obtained with the *eyFlp*-*cell lethal* system. Scale bars represent 100 µm. (A) Control eye-antennal imaginal disc. (B–D) Eye-antennal imaginal discs predominantly mutant for *vps22* (B), *vps25* (C) and *vps36* (D). Genotypes: (A) *eyFlp* ; *FRT82B*/*FRT82B cl GMR*-*hid*. (B) *eyFlp* ; *FRT82B vps22^5F8-3^*/*FRT82B cl GMR*-*hid*. (C) *eyFlp* ; *FRT42 vps25^N55^*/*FRT42 cl GMR*-*hid*. (D) *eyFlp* ; *vps36^L5212^ FRT80*/*GMR*-*hid cl FRT80*.

After establishing that ESCRT-II mutants have similar endosomal defects, we analyzed them for the presence of neoplastic and hyperplastic growth phenotypes. Neoplastic growth phenotypes have been observed for *vps25*, if almost the entire disc is mutant [20], [26]. Eye discs predominantly mutant for a gene were obtained using the *eyFlp-cell lethal* system [26], [41]. In this system, all cells which are not mutant for the gene of interest are eliminated by homozygosity of the *cell lethal* mutation or by induction of apoptosis using *GMR-hid* [41]. When we applied this technique to ESCRT-II mutants, the resulting mutant eye discs are overgrown compared to normal discs (Fig. 4). This overgrowth is particularly striking for *vps25* mutant discs, consistent with previous reports [26], but also *vps22* and *vps36* mutant discs are significantly larger than normal discs (Fig. 4).

We labeled these discs with phalloidin, a marker for cortical actin. In wild-type discs, phalloidin labeling reveals regular cellular architecture (Fig. 4A). However, discs predominantly mutant for *vps22*, *vps25* and *vps36* show disorganized cellular architecture. Similar observations have been made for other neoplastic tumor suppressor genes including *ept* (*vps23*), *avalanche*, *rab5* and *lgl* [26]. Therefore, *vps22*, *vps25* and *vps36* mutant discs display neoplastic tumor characteristics.

### Distinct phenotypes I: *vps22* and *vps25* mosaics, but not *vps36*, display non-autonomous overgrowth

**Figure 5:**
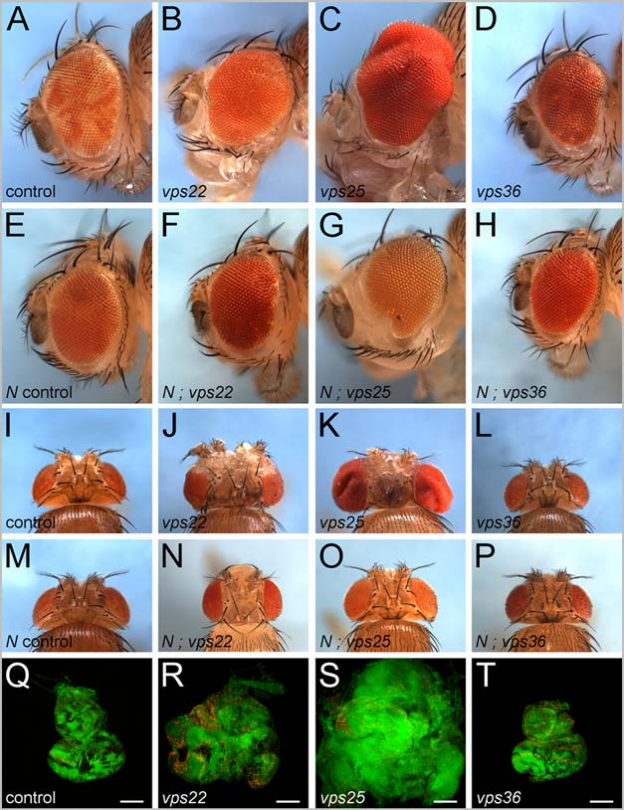
Adult phenotypes of ESCRT-II mosaics. *vps22* and *vps25* mosaics display strong overgrowth phenotypes of the adult eyes and heads, and the larval eye imaginal discs. In contrast, *vps36* mutants show no or only a mild proliferation phenotype and cause a roughening of the adult eye. (A–D) Side view of genetic eye mosaics of (A) control flies, (B) *vps22^5F8-3^*, (C) *vps25^N55^* and (D) *vps36^L5212^* mutants. (E–H) Eye mosaics of (E) control (heterozygous *Notch*), (F) *vps22^5F8-3^*, (G) *vps25^N55^* and (H) *vps36^L5212^* in heterozygous *Notch* (*N*) background. The Notch allele used is *N^264-39^*. (I–L) Top view of genetic mosaics of (I) control flies, (J) *vps22^5F8-3^*, (K) *vps25^N55^* and (L) *vps36^L5212^* mutants. (M–P) Head mosaics of (M) control (heterozygous *Notch*), (N) *vps22^5F8-3^*, (O) *vps25^N55^* and (P) *vps36^L5212^* in heterozygous *Notch* (*N*) background. The Notch allele used is *N^264-39^*. (Q–T) Size comparison of (Q) control, (R) *vps22^5F8-3^*, (S) *vps25^N55^* and (T) *vps36^L5212^* mosaic eye imaginal discs. Green: GFP; red: BrdU labeling. The scale bars represent 100 µm. Genotypes: (A) *eyFlp* ; *FRT82B/FRT82B P[w^+^]*. (B,J) *eyFlp* ; *FRT82B vps22^5F8-3^/FRT82B P[w^+^]*. (C,K) *eyFlp* ; *FRT42D vps25^N55^/FRT42D P[w^+^]*. (D,L) *eyFlp* ; *vps36^L5212^ FRT2A/P[w^+^] FRT2A*. (E,M) *N^264-39^*/+. (F–H) and (N–P): same as (B–D) and (J–L) except they also carry *N^264-39^* as heterozygous mutation. (Q–T) same as in corresponding panels A–D except they carry P[ubi-GFP] instead of P[w^+^].

Next, we tested whether *vps22* and *vps36* mosaics - similar to *vps25* - cause non-autonomous overgrowth which is the result of increased Notch signaling activity [19]–[21]. In Figure 2, we showed that *vps22* and *vps36* mutant clones contain increased Notch protein levels. Therefore, we expected that *vps22* and *vps36* mosaic animals would display the same non-autonomous overgrowth phenotype as *vps25* mosaics (Fig. 5C,K). Surprisingly, that was only observed for *vps22* mosaics, but not for *vps36* mosaics. Eyes and heads of *vps22* mosaics are significantly larger compared to wild-type controls (Fig. 5A,B,I,J). The overgrowth is also detectable in eye-antennal imaginal discs, the larval precursors of the adult eyes. *vps22* mosaic eye-antennal discs are significantly larger compared to wild-type controls (Fig. 5Q,R). This overgrowth is non-autonomous because, as shown in Figure 3, *vps22* mutant clones are apoptotic and cannot be recovered in mosaic *vps22* eyes (mutant *vps22* tissue is marked by the absence of red eye pigment, i.e. are phenotypically *white* ^−^; note the lack of *white* ^−^ tissue in Fig. 5B compared to the wild-type control in Fig. 5A).

Surprisingly, a similar strong overgrowth phenotype was not found for *vps36* mosaic eyes and heads (Fig. 5D,L). We also found this confirmed at the level of eye imaginal discs. *vps36* mosaic eye discs are noticeably smaller than *vps22* and *vps25* mosaic discs and are comparable in size to wild-type (Fig. 5Q–T). We do note, however, a rough eye phenotype in *vps36* mosaics (Fig. 5D). Thus, although *vps22*, *vps25* and *vps36* mutants display similar endosomal defects and contain increased Notch protein levels, they affect mosaic animals differently.

Because we observed increased Notch protein levels in *vps22*, *vps25* and *vps36* mutant clones (Fig. 2), we analyzed whether Notch accounts for the overgrowth phenotypes in *vps22* and *vps25* mosaics. This can be tested by determining whether the overgrowth phenotype can be suppressed by reducing the gene dose of *Notch*. Thus, we analyzed *vps22*, *vps25* and *vps36* mosaics in a heterozygous *Notch* background. Indeed, heterozygosity of *Notch* suppresses the overgrowth of *vps22* and *vps25* eyes and heads suggesting that Notch activity is required for the overgrowth phenotype of *vps22* and *vps25* mosaics (Fig. 5E–G, M–O). The rough eye phenotype observed for *vps36* mosaics is not suppressed by heterozygous *Notch* (Fig. 5D,H).

**Figure 6:**
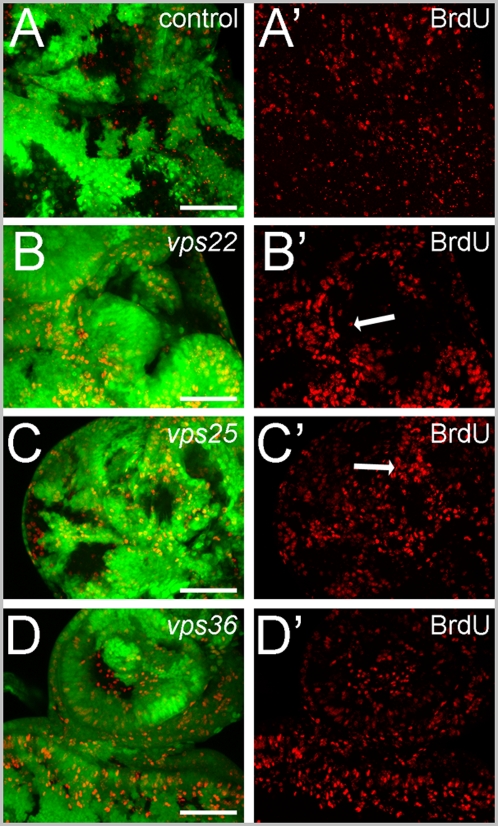
Proliferation phenotype of ESCRT-II mosaics. Non-autonomous regulation of proliferation in *vps22* and *vps25* eye mosaics as depicted by BrdU incorporation (red). Arrows in (B) and (C) point to areas of increased BrdU density next to mutant clones. Compared to control discs, *vps36* mutations do not affect the proliferation pattern significantly. The scale bar represents 50 µm. (A–D) GFP/BrdU (green/red) co-labelings of (A) control, (B) *vps22^5F8-3^*, (C) *vps25^N55^* and (D) *vps36^L5212^* eye mosaics. (A′–D′) BrdU labeling of (A′) control, (B′) *vps22^5F8-3^*, (C′) *vps25^N55^* and (D′) *vps36^L5212^* eye mosaics. Genotypes: (A) *eyFlp*; *FRT42B/FRT42B P[ubi-GFP]*. (B) *eyFlp*; *FRT82B vps22^5F8-3^/FRT82B P[ubi-GFP]*. (C) *eyFlp*; *FRT42D vps25^N55^/FRT42D P[ubi-GFP]*. (D) *eyFlp*; *vps36^L5212^ FRT2A/P[ubi-GFP] FRT2A*.

To further characterize these differences in the overgrowth phenotypes, we analyzed 3^rd^ instar eye-antennal imaginal discs of the ESCRT-II mutants by BrdU labeling as a marker for cells in S-phase. Anterior to the morphogenetic furrow and in the antennal disc, BrdU-labeling is homogeneous in wild-type discs (Fig. 6A). In contrast, *vps22* and *vps25* mutant clones do not proliferate very well (Fig. 6B,C). However, the wild-type tissue immediately adjacent to *vps22* mutant clones shows increased density of BrdU-positive cells (compare Fig. 6B′,C′ to wild-type in Fig. 6A′). Thus, similar to *vps25* mosaics, *vps22* controls cell proliferation non-autonomously. However, *vps36* mosaics behave differently. We observe BrdU-positive cells both within and outside of *vps36* clones with homogeneous density (Fig. 6D′). There is no apparent increased density of BrdU-positive cells outside of *vps36* mutant clones. Thus, *vps36* mutant clones appear to be unable to induce non-autonomous proliferation.

### Distinct phenotypes II: *vps22* and *vps25* mutant clones, but not *vps36*, contain strong Notch activity

The analyses presented above demonstrated that mutants of the three ESCRT-II components display endosomal defects with accumulated Notch protein (Figures 1 and 2); yet, only *vps22* and *vps25* mutant clones trigger non-autonomous proliferation in a Notch-dependent manner, while *vps36* clones do not (Figures 5 and 6). One possibility to explain this discrepancy is that only *vps22* and *vps25*, but not *vps36* mutations trigger Notch signaling although Notch protein accumulates in all three mutants (Figure 2). Therefore, we assayed for Notch activity in ESCRT-II mutants using the *E(spl)m8 2.61-lacZ* reporter transgene that responds well to Notch activity [21].

**Figure 7:**
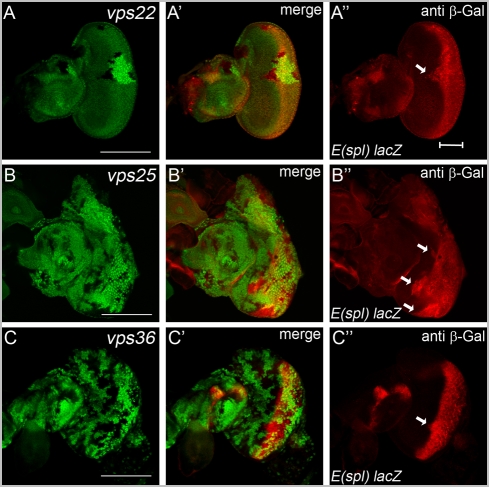
Notch activity in ESCRT-II mutants. Notch activity in ESCRT-II mutants was assessed using the reporter transgene *E*(*spl*)*m8 2.61*-*lacZ* and β-Gal immunohistochemistry. In wild-type discs, this reporter is turned on posterior to the morphogenetic furrow (see bar in A″). Note that in *vps22^5F8-3^* and *vps25^N55^* mutant clones located anterior to the morphogenetic furrow ectopic reporter activity is detectable (arrows in A″ and B″). *vps36^L5212^* clones do not or only weakly (arrow) induce reporter activity (C″). Genotypes: (A) *eyFlp*; *E*(*spl*)*m8 2.61*-*lacZ* ; *FRT82B vps22^5F8-3^/FRT82B P[w^+^]*. (B) *eyFlp* ; *FRT42D vps25^N55^ E*(*spl*)*m8 2.61*-*lacZ/FRT42D E*(*spl*)*m8 2.61-lacZ*. (C) *eyFlp* ; *E*(*spl*)*m8 2.61*-*lacZ*; *vps36^L5212^ FRT2A/P[w^+^] FRT2A*.

The *E(spl)m8 2.61-lacZ* reporter is turned on by normal (wild-type) Notch activity posterior to the morphogenetic furrow in eye imaginal discs (Fig. 7A,C). There is no reporter activity detectable anterior to the morphogenetic furrow. However, in *vps22* and *vps25* mutant clones located anterior to the morphogenetic furrow, *E(spl)m8 2.61-lacZ* reporter expression can be detected (arrows in Fig. 7A″ and B″). In contrast, clones of *vps36* located anterior to the morphogenetic furrow do not or only very mildy increase reporter activity (Figure 7C″). This behavior was consistently observed in fifteen eye imaginal discs of each genotype. Thus, the observed differences in non-autonomous overgrowth between *vps22*, *vps25* and *vps36* do correlate with de-regulation of Notch activity.

### Distinct phenotypes III: *vps25* and *vps36* mutations, but not *vps22*, promote strong apoptotic resistance

**Figure 8:**
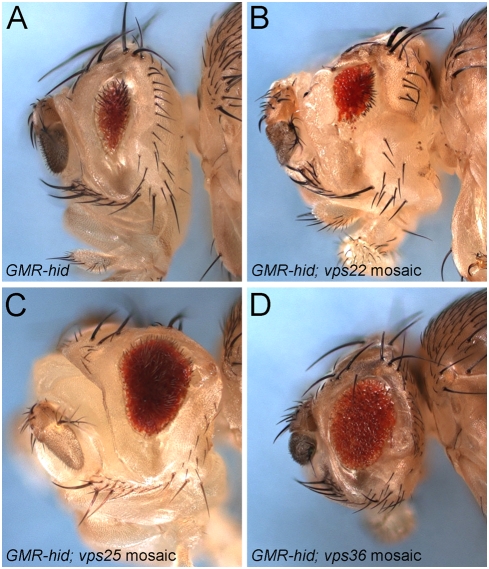
Suppression of the *GMR-hid*-eye ablation phenotype by ESCRT-II mosaics. (A) Expression of the pro-apoptotic gene *hid* under control of the eye-specific *GMR* enhancer (*GMR-hid*) gives rise to a strong eye ablation phenotype due to excessive apoptosis. (B–D) *vps25^N55^* (C) and *vps36^L5212^* (D) eye mosaics are strong suppressors of the *GMR-hid*-induced eye ablation phenotype in adult flies. *vps22^5F8-3^* mosaics (B) do not suppress the *GMR-hid*-eye ablation phenotype. Genotypes: (A) *eyFlp ; GMR-hid*; *FRT82B/FRT82B P[w^+^]*. (B) *eyFlp*; *GMR-hid*; *FRT82B vps22^5F8-3^/FRT82B P[w^+^]*. (C) *GMR-hid eyFlp*; *FRT42D vps25^N55^/FRT42D P[w^+^]*. (D) *eyFlp*; *GMR-hid*; *vps36^L5212^ FRT2A/P[w^+^] FRT2A*.

Paradoxically, despite the fact that *vps25* clones are highly apoptotic (Figure 3B), we originally isolated *vps25* mutants based on their ability to suppress apoptosis [21]. Specifically, the eye-ablation phenotype caused by expression of the pro-apoptotic gene *hid* under control of the eye-specific *GMR* promoter (*GMR*-*hid*) is suppressed in *vps25* mosaics (see Fig. 8A,C). The suppression of *GMR-hid* by *vps25* occurs in a non-autonomous manner through up-regulation of the apoptosis inhibitor Diap1 in neighboring cells [21]. Therefore, we tested whether *vps22* and *vps36* have a similar activity. However, to our surprise, although *vps22* mosaics cause a strong non-autonomous proliferation phenotype, they do not suppress *GMR*-*hid* (Fig. 8B). In contrast, although *vps36* mosaics display no or only a mild non-autonomous proliferation phenotype, they are very strong suppressors of *GMR*-*hid*, comparable to *vps25* mosaics (Fig. 8D). This observation provides another example of genetic differences between the individual components of ESCRT-II.

## Discussion

In this paper, we have characterized and compared the mutant phenotypes of the ESCRT-II components *vps22*, *vps25* and *vps36* in *Drosophila*. So far, a systematic genetic analysis of class E *vps* genes has only been performed in yeast [12], [15]. Endosomal defects in these mutants in yeast are genetically similar. Consistently, endosomal defects caused by mutations in the ESCRT-II components *vps22*, *vps25* and *vps36* in *Drosophila* are similar, too. These mutant endosomes accumulate ubiquitinated proteins and signaling receptors including Notch and its ligand Delta. They also show neoplastic characteristics. However, despite these common endosomal defects, at the organismal level, *vps22*, *vps25* and *vps36* mosaic animals display distinct phenotypes. *vps22* mosaics are characterized by strong non-autonomous proliferation, but not an increase in apoptotic resistance. *vps36* mosaics exhibit the reverse phenotype, i.e. increased apoptotic resistance and no or only weak non-autonomous proliferation. As shown before [21], *vps25* mosaics combine both phenotypes. Thus, this analysis shows that although these components are part of the same structural complex, they are not genetically equivalent and display distinct genetic properties.

While the *vps22* allele used in this study is a clear null allele [32], one might argue that the *vps36* allele is not a null and that the observed differences are due to the hypomorphic nature of *vps36*. However, such an assumption does not explain why *vps36* is a strong suppressor of *GMR*-*hid*, while a null allele of *vps22* that causes a strong overgrowth phenotype, completely fails to suppress *GMR*-*hid* (Fig. 8B). In addition, the common phenotypes (endosomal defects giving rise to enlarged endosomes, accumulation of Notch protein, apoptosis and the neoplastic phenotype) are very similar between *vps22* and *vps36*. Thus, it does not appear that the phenotypic differences observed between *vps22* and *vps36* are due to allelic strength of the mutants. Rather, they appear to be caused by intrinsic differences of the endogenous genes.

### Role of Notch signaling for non-autonomous hyperplastic proliferation

It has previously been shown that inappropriate Notch signaling is required for non-autonomous proliferation in *vps25* mosaics [18]–[21]. Our data confirm this notion here for *vps22* mosaics. *vps22* and *vps25* mutants contain increased Notch activity and heterozygosity of *Notch* suppresses the non-autonomous overgrowth phenotype. In contrast, *vps36* mosaics do not activate Notch signaling and hence do not cause non-autonomous overgrowth. Thus, Notch activity is required for non-autonomous hyperplastic overgrowth.

It is puzzling that despite their intimate physical association in the ESCRT-II complex [33], [34], loss of *vps22*, *vps25* and *vps36* affects Notch signaling differently. One possibility to explain these differences is that these mutants form distinct endosomal microenvironments which may affect signaling from the endosome differently. The resolution of our labeling technologies may not be sufficient to pick up these differences in the endosomal microenvironment, but the fact that we do observe genetic differences suggests that microenvironmental differences may exist. There is precedence for such a conclusion. Although *hrs* mutants contain abnormal endosomes leading to accumulation of Notch protein, they do not trigger Notch activity and hence no significant growth defects [16]. Further support of the idea that Notch needs to be in a particular microenvironment at the early endosome in order to be activated comes from a study that analyzes that act upstream of the ESCRT machinery in the endosomal pathway, namely *shibire*, *avalanche* and *Rab5*. Mutations in these genes also result in accumulation of Notch protein, but do not activate the pathway [8].

Class E *vps* genes have also been reported to function outside of endosomal protein sorting. As such they are involved in virus budding, transcriptional control, cell cycle progression, mRNA localization and apoptosis [31], [32], [42]–[48]. Therefore, it is possible that the observed genetic differences of the ESCRT-II components may be caused by distinct requirements in addition to and independently of endosomal function and possibly independently of the ESCRT-II complex and the remaining ESCRT machinery. Future work will be necessary to dissect the roles of the ESCRT-II components in processes unrelated to endosomal processing.

### Role of Notch signaling for autonomous neoplastic proliferation

While inappropriate Notch signaling correlates well with non-autonomous hyperplastic growth, it does not correlate with autonomous neoplastic growth. Imaginal discs entirely mutant for *vps22*, *vps25* and *vps36* all display overgrowth and loss of cellular architecture, hallmarks of neoplastic behavior [22]. The neoplastic phenotype has been attributed to either increased Notch signaling or to mis-localization of the apical transmembrane protein Crumbs [49]. However, *vps36* mutant discs display a very robust neoplastic phenotype, but do not activate the Notch signaling pathway significantly, suggesting that activation of Notch is not required for neoplastic growth in *vps36* mutant discs. This observation is consistent with previous findings that mutations in the neoplastic tumor suppressor genes *avalanche* and *Rab5* do not activate Notch signaling [8], [49]. We have not analyzed a genetic requirement of *crumbs* for the neoplastic phenotype in *vps22*, *vps25* and *vps36* mutants, but that would be an interesting experiment in the future.

It is clear that the endosomal defects in ESCRT-II mutants not only affect Notch signaling. Other membrane proteins are also affected which may contribute to the neoplastic phenotype. For example, in the case of *hrs* and *vps25*, other signaling receptors such as EGFR, Tkv, Ptc and Smo accumulate at endosomes [16], [19]. However, it was also shown that these accumulated proteins are largely derived from the pool of unliganded receptors, suggesting that the endosomal defect affects receptor turnover [16] which does not necessarily cause receptor activation. The only receptor known to be activated at the endosome in a ligand-independent manner is Notch [8]. Future work will be necessary to dissect the role of Crumbs and other signaling pathways for developing the neoplastic phenotypes.

## Materials and Methods

### *Drosophila* genetics and generation of mutant clones

For this comparative analysis, we used the following mutant alleles of the ESCRT-II components. *vps22^5F8^* ^-3^ (also known as *lsn^5F8^* ^-3^) was previously described by Irion and St. Johnston (2007) [32]. It carries a premature termination codon at residue 2, likely encoding a null allele. *vps25^N55^* has a premature termination codon at residue 93. We have previously characterized this allele as a null allele [21]. *vps36^L5212^* was also characterized by Irion and St Johnston (2007) [32]. A P-element transposon is inserted in the first exon 29 base pairs upstream of the initiator ATG.

Fly crosses were conducted using standard procedures at 25°C. The following stocks were used: *vps22^5F8-3^* and *vps36^L5212^* [32]; *vps25^N55^* [21]; *N^264-39^* [50]; *GMR-hid eyFlp* [51]-[53]. For generation of mutant clones, the *vps* mutant alleles were crossed to *eyFlp*; FRT P[*ubi-GFP*]. To generate imaginal discs predominantly mutant for *vps22*, *vps25* or *vps36*, we used the *eyFlp cell lethal* technique [41]. The *vps* mutant alleles were crossed to *eyFlp*; *FRT cl GMR-hid* flies. *cl* indicates an anonymous cell lethal mutation that kills when homozygous [41]. The use of the FRT depended on the location of the *vps* gene in the genome. Mutant clones are marked by absence of GFP. The complete genotypes are indicated in the legend to the figures.

### Immunohistochemistry

Eye imaginal discs from 3^rd^ instar larvae were dissected and immunohistochemical labeling was performed as described [21]. The following antibodies were used: anti-Hrs (kind gift of Hugo Bellen); FK1 and FK2 (Biomol International); anti-Ubiquitin (Sigma); anti-N[intra], anti-N[extra], anti-Delta and anti-β-Gal (DSHB, University of Iowa); anti-BrdU (Becton Dickinson); anti-cleaved caspase-3 (Cell Signaling Technology), and TRITC-phalloidin (Sigma-Aldrich). Cy3- and Cy-5 fluorescently-conjugated secondary antibodies are obtained from Jackson ImmunoResearch. Images were captured using Olympus Optical FV500 or FV1000 confocal microscopes.

## References

[1] Haglund K, Dikic I (2005). Ubiquitylation and cell signaling.. Embo J.

[2] Hicke L, Schubert HL, Hill CP (2005). Ubiquitin-binding domains.. Nat Rev Mol Cell Biol.

[3] Williams RL, Urbe S (2007). The emerging shape of the ESCRT machinery.. Nat Rev Mol Cell Biol.

[4] Gonzalez-Gaitan M (2003). Signal dispersal and transduction through the endocytic pathway.. Nat Rev Mol Cell Biol.

[5] Le Roy C, Wrana JL (2005). Signaling and endocytosis: a team effort for cell migration.. Dev Cell.

[6] Seto ES, Bellen HJ (2004). The ins and outs of Wingless signaling.. Trends Cell Biol.

[7] Seto ES, Bellen HJ (2006). Internalization is required for proper Wingless signaling in Drosophila melanogaster.. J Cell Biol.

[8] Vaccari T, Lu H, Kanwar R, Fortini ME, Bilder D (2008). Endosomal entry regulates Notch receptor activation in Drosophila melanogaster.. J Cell Biol.

[9] Babst M (2005). A protein's final ESCRT.. Traffic.

[10] Felder S, Miller K, Moehren G, Ullrich A, Schlessinger J (1990). Kinase activity controls the sorting of the epidermal growth factor receptor within the multivesicular body.. Cell.

[11] Gruenberg J, Stenmark H (2004). The biogenesis of multivesicular endosomes.. Nat Rev Mol Cell Biol.

[12] Katzmann DJ, Odorizzi G, Emr SD (2002). Receptor downregulation and multivesicular-body sorting.. Nat Rev Mol Cell Biol.

[13] Odorizzi G, Babst M, Emr SD (1998). Fab1p PtdIns(3)P 5-kinase function essential for protein sorting in the multivesicular body.. Cell.

[14] Raiborg C, Rusten TE, Stenmark H (2003). Protein sorting into multivesicular endosomes.. Curr Opin Cell Biol.

[15] Raymond CK, Howald-Stevenson I, Vater CA, Stevens TH (1992). Morphological classification of the yeast vacuolar protein sorting mutants: evidence for a prevacuolar compartment in class E vps mutants.. Mol Biol Cell.

[16] Jekely G, Rorth P (2003). Hrs mediates downregulation of multiple signalling receptors in Drosophila.. EMBO Rep.

[17] Lloyd TE, Atkinson R, Wu MN, Zhou Y, Pennetta G (2002). Hrs regulates endosome membrane invagination and tyrosine kinase receptor signaling in Drosophila.. Cell.

[18] Moberg KH, Schelble S, Burdick SK, Hariharan IK (2005). Mutations in erupted, the Drosophila ortholog of mammalian tumor susceptibility gene 101, elicit non-cell-autonomous overgrowth.. Dev Cell.

[19] Thompson BJ, Mathieu J, Sung HH, Loeser E, Rorth P (2005). Tumor suppressor properties of the ESCRT-II complex component Vps25 in Drosophila.. Dev Cell.

[20] Vaccari T, Bilder D (2005). The Drosophila tumor suppressor vps25 prevents nonautonomous overproliferation by regulating notch trafficking.. Dev Cell.

[21] Herz HM, Chen Z, Scherr H, Lackey M, Bolduc C (2006). vps25 mosaics display non-autonomous cell survival and overgrowth, and autonomous apoptosis.. Development.

[22] Hariharan IK, Bilder D (2006). Regulation of imaginal disc growth by tumor-suppressor genes in Drosophila.. Annu Rev Genet.

[23] Chao JL, Tsai YC, Chiu SJ, Sun YH (2004). Localized Notch signal acts through eyg and upd to promote global growth in Drosophila eye.. Development.

[24] Reynolds-Kenneally J, Mlodzik M (2005). Notch signaling controls proliferation through cell-autonomous and non-autonomous mechanisms in the Drosophila eye.. Dev Biol.

[25] Tsai YC, Sun YH (2004). Long-range effect of upd, a ligand for Jak/STAT pathway, on cell cycle in Drosophila eye development.. Genesis.

[26] Menut L, Vaccari T, Dionne H, Hill J, Wu G (2007). A mosaic genetic screen for Drosophila neoplastic tumor suppressor genes based on defective pupation.. Genetics.

[27] Li L, Cohen SN (1996). Tsg101: a novel tumor susceptibility gene isolated by controlled homozygous functional knockout of allelic loci in mammalian cells.. Cell.

[28] Li L, Li X, Francke U, Cohen SN (1997). The TSG101 tumor susceptibility gene is located in chromosome 11 band p15 and is mutated in human breast cancer.. Cell.

[29] Lin SY, Chen YJ, Chang JG (1998). Multiple truncated transcripts of TSG101 in gastrointestinal cancers.. J Gastroenterol Hepatol.

[30] Sun Z, Pan J, Bubley G, Balk SP (1997). Frequent abnormalities of TSG101 transcripts in human prostate cancer.. Oncogene.

[31] Krempler A, Henry MD, Triplett AA, Wagner KU (2002). Targeted deletion of the Tsg101 gene results in cell cycle arrest at G1/S and p53-independent cell death.. J Biol Chem.

[32] Irion U, St Johnston D (2007). bicoid RNA localization requires specific binding of an endosomal sorting complex.. Nature.

[33] Teo H, Perisic O, Gonzalez B, Williams RL (2004). ESCRT-II, an endosome-associated complex required for protein sorting: crystal structure and interactions with ESCRT-III and membranes.. Dev Cell.

[34] Hierro A, Sun J, Rusnak AS, Kim J, Prag G (2004). Structure of the ESCRT-II endosomal trafficking complex.. Nature.

[35] Fujimuro M, Sawada H, Yokosawa H (1994). Production and characterization of monoclonal antibodies specific to multi-ubiquitin chains of polyubiquitinated proteins.. FEBS Lett.

[36] Fujimuro M, Sawada H, Yokosawa H (1997). Dynamics of ubiquitin conjugation during heat-shock response revealed by using a monoclonal antibody specific to multi-ubiquitin chains.. Eur J Biochem.

[37] Lee TV, Ding T, Chen Z, Rajendran V, Scherr H (2008). The E1 ubiquitin-activating enzyme Uba1 in Drosophila controls apoptosis autonomously and tissue growth non-autonomously.. Development.

[38] Yu SY, Yoo SJ, Yang L, Zapata C, Srinivasan A (2002). A pathway of signals regulating effector and initiator caspases in the developing Drosophila eye.. Development.

[39] Fan Y, Bergmann A (2008). Apoptosis-induced compensatory proliferation. The Cell is dead. Long live the Cell!. Trends Cell Biol.

[40] Fan Y, Bergmann A (2008). Distinct mechanisms of apoptosis-induced compensatory proliferation in proliferating and differentiating tissues in the Drosophila eye.. Dev Cell.

[41] Stowers RS, Schwarz TL (1999). A genetic method for generating Drosophila eyes composed exclusively of mitotic clones of a single genotype.. Genetics.

[42] de Gassart A, Geminard C, Hoekstra D, Vidal M (2004). Exosome secretion: the art of reutilizing nonrecycled proteins?. Traffic.

[43] Demirov DG, Freed EO (2004). Retrovirus budding.. Virus Res.

[44] Kamura T, Burian D, Khalili H, Schmidt SL, Sato S (2001). Cloning and characterization of ELL-associated proteins EAP45 and EAP20. a role for yeast EAP-like proteins in regulation of gene expression by glucose.. J Biol Chem.

[45] Pornillos O, Garrus JE, Sundquist WI (2002). Mechanisms of enveloped RNA virus budding.. Trends Cell Biol.

[46] Schmidt AE, Miller T, Schmidt SL, Shiekhattar R, Shilatifard A (1999). Cloning and characterization of the EAP30 subunit of the ELL complex that confers derepression of transcription by RNA polymerase II.. J Biol Chem.

[47] Wagner KU, Krempler A, Qi Y, Park K, Henry MD (2003). Tsg101 is essential for cell growth, proliferation, and cell survival of embryonic and adult tissues.. Mol Cell Biol.

[48] Slagsvold T, Pattni K, Malerod L, Stenmark H (2006). Endosomal and non-endosomal functions of ESCRT proteins.. Trends Cell Biol.

[49] Lu H, Bilder D (2005). Endocytic control of epithelial polarity and proliferation in Drosophila.. Nat Cell Biol.

[50] de Celis JF, Mari-Beffa M, Garcia-Bellido A (1991). Cell-autonomous role of Notch, an epidermal growth factor homologue, in sensory organ differentiation in Drosophila.. Proc Natl Acad Sci U S A.

[51] Grether ME, Abrams JM, Agapite J, White K, Steller H (1995). The head involution defective gene of Drosophila melanogaster functions in programmed cell death.. Genes Dev.

[52] Xu D, Li Y, Arcaro M, Lackey M, Bergmann A (2005). The CARD-carrying caspase Dronc is essential for most, but not all, developmental cell death in Drosophila.. Development.

[53] Srivastava M, Scherr H, Lackey M, Xu D, Chen Z (2007). ARK, the Apaf-1 related killer in Drosophila, requires diverse domains for its apoptotic activity.. Cell Death Differ.

